# Lifestyle and Sociodemographic Risk Factors for Death among Middle-aged and Elderly Residents in Japan from a Five-year Follow-up Cohort Study

**DOI:** 10.2188/jea.11.51

**Published:** 2007-11-30

**Authors:** Akiko Ohta, Shigenobu Aoki, Kazuo Takeuchi, Sasazawa Yosiaki, Shosuke Suzuki

**Affiliations:** 1 Department of Public Health, Gunma University School of Medicine.; 2 Faculty of Social and Information Studies, Gunma University.; 3 Gunma Prefectural College of Health Sciences.

**Keywords:** prospective study, mortality, lifestyle, body mass index, perceived health status

## Abstract

To examine the relationship between lifestyle and sociodemographic risk factors and mortality, a population-based prospective cohort study was conducted in two areas of Gunma Prefecture, Japan, and a cohort consisting of 11,565 subjects aged 40-69 at baseline in 1993 was followed. During the five-year follow-up period, 201 men and 103 women died. The relative risks (RRs) of risk factors were estimated by the Cox proportional-hazards model. Significant RRs with multivariate adjustment for all-cause mortality was observed for body mass index (BMI). The curve for the relationship between BMI and all-cause mortality was L-shaped in men and U-shaped in women, with the lowest RRs at a BMI of 22-25 in both men and women. Other significant RRs for all-cause mortality were observed for obesity in the subjects’ 30’s in both men and women (RR: 2.42 and RR: 2.75), poor perceived health status in men (RR: 4.55), and having had a health examination in the past three years in both men and women (RR: 0.49 and RR: 0.46). These results suggested that increased risk of death was independently associated with a lower BMI, obesity in the subjects’ 30’s, and not undergoing health examinations, among both men and women, and poor perceived health status among men.

## INTRODUCTION

The importance of the association between lifestyle and psychosocial factors and mortality has been reported in several cohort studies, for example, the Framingham Study^1^^)^ and the Alameda County Study^2^^, ^^3^^)^. Since most of the studies have been conducted in Western countries, their findings may not be directly applicable to Japanese people, because the prevalence of disease, race, lifestyle, and socioenvironmental factors in Japan differ from those in Western countries.

During the past 30 years, prospective cohort studies based on residents of communities in Japan have mainly focused on physical health as risk factors of mortality^4^^)^, but recently the effects of lifestyle and psychosocial factors on mortality also have been reported^5^^-^^7^^)^. More studies on lifestyle and sociodemographic factors as well as physical data are still needed on Japanese community population.

Our study was designed as a population-based prospective cohort study to examine the association between lifestyle, psychosocial, and sociodemographic factors, and mortality. We conducted a baseline questionnaire survey of all residents aged 40-69 years in Komochi Village and Isesaki City, Gunma Prefecture, Japan in 1993. The authors have already reported some of the results obtained from the baseline data^8^^-^^10^^)^. In this paper we report the effects of lifestyle and sociodemographic factors on all-cause mortality, based on survival status during a five-year follow-up period.

## MATERIALS AND METHODS

### Subjects

The subjects were all of the residents aged 40-69 years of Komochi Village and the downtown area of Isesaki City, identified in the Basic Resident Registers of Komochi Village as of September 1992 and of Isesaki City as of August 1993.

Both areas are located in the center of Gunma Prefecture, Japan. In 1995, the population of Komochi Village and Isesaki City was 12,141 and 120,236, respectively. In 1995, primary, secondary, and tertiary industry workers accounted for 16.4%, 36.5%, and 47.1%, respectively, of the employed persons in Komochi Village, and in Isesaki City, the respective percentages were 3.7%, 44.8%, and 51.5%.

### Baseline survey

Self-administered questionnaires were distributed through the respective municipal government offices to all of the residents of Komochi Village in January 1993 and to those of the downtown area of Isesaki City in October 1993, and completed questionnaires in sealed envelopes were collected. The questionnaire consisted of items on sociodemographic characteristics, health status, lifestyle, social network, and the Todai Health Index (THI)^11^^)^, which is a symptom check list that quantitatively assesses physical and mental complaints. A total of 4,501 residents responded in Komochi Village (response rate 92.3%) and of 7,064 residents responded in Isesaki City (response rate 91.1%). A total of 11,565 residents (5,630 men and 5,935 women) in both areas responded to the questionnaire (response rate 91.6%) on the baseline survey. Non-respondents were not recontacted.

### Follow-up survey

We researched the survival status of subjects totalling 11,565 persons from 1993 to 1998. Deaths and migrations were identified in the municipal Basic Resident Registers of each area. Causes of death were identified by death certificates at the public health center in each area with the permission of the Management and Coordination Agency of Japan. In 1997 and 1998, we added a mail inquiry as a means of reaching subjects who had moved away from the study areas. Subjects who did not respond to the mail inquiry or who had not been reached were regarded as censored cases. Causes of death were coded according to the International Classification of Diseases, Tenth Revision (ICD-10).

### Risk Factor Measures

We calculated body mass index (BMI; weight in kilograms divided by the square of height in meters) from self-reported height and weight. Smoking status was assessed by asking, “Do you smoke cigarettes: never, former, or current smoker?” Current smokers were also asked the number of cigarettes smoked per day. Alcohol drinking was assessed by asking, “Do you drink a lot of alcoholic beverages: yes, drink a little, or never?” Physical activity was assessed by asking, “Do you exercise regularly: often, sometimes, or never?” Hours of sleep, snacking between meals (often, sometimes, or never), and skipping breakfast (often, sometimes, or never) were also assessed. Weight in the subjects’ 30’s was assessed by asking, “How was your body weight in your 30’s: underweight, average weight, or overweight?”

Perceived health status was assessed by asking, “What is your current health condition: excellent, good, fair, poor, or very poor?” The subjects were also asked about the presence of chronic diseases (yes or no) and having had a health examination in the past three years (yes or no). The sociodemographic factors asked were sex, age, longest occupation, marital status, and educational background.

### Statistical Analysis

We analyzed the associations between risk factors and all-cause mortality based on survival status at the end of March 1998. The Cox proportional-hazards model^12^^)^ was used to compute relative risks (RRs) with 95% confidence intervals (CIs).

Explanatory variables for these analyses were as follows: age, BMI, smoking status, alcohol drinking, physical activity, hours of sleep, snacking between meals, skipping breakfast, weight in the subjects’ 30’s, perceived health status, chronic diseases, health examination in the past three years, longest occupation, marital status, and educational background. In the analysis, BMI was coded into five categories: less than 19, 19 to less than 22, 22 to less than 25, 25 to less than 27, and 27 or more. Smoking status was coded into four categories: never, former, current smoker of fewer than 20 cigarettes per day, and current smoker of 20 or more cigarettes per day. Hours of sleep were coded into three categories: less than 7 hours, 7-8 hours, and more than 8 hours. Other variables were coded as shown in Table 4.

Age-adjusted RRs were calculated by sex, and multivariate RRs adjusted for all of the variables listed above and the variable for the study areas (Komochi Village and Isesaki City) were then calculated by sex. Statistical analyses were performed with the statistical package SPSS 9.0J for Windows.

## RESULTS

### Baseline characteristics

The mean (± standard deviation [SD]) age of the male subjects was 54.0 (± 8.5) years, and 54.5 (± 8.4) years for the women. The distribution of the variables included in the analysis is shown Table 1 and Table 2.

**Table 1.** Sociodemographic characteristics at the time of the baseline survey and the numbers of deaths of men and women.

Characteristic	Men				Women			
	
	N	%	No. of deaths	%	N	%	No. of deaths	%
Age class								
40-44 years	1044	18.5	7	3.5	952	16.0	3	2.9
45-49 years	945	16.8	13	6.5	996	16.8	11	10.7
50-54 years	947	16.8	23	11.4	947	16.0	10	9.7
55-59 years	885	15.7	32	15.9	1086	18.3	20	19.4
60-64 years	1053	18.7	51	25.4	1069	18.0	28	27.2
65-69 years	756	13.4	75	37.3	885	14.9	31	30.1
Occupation								
Salaried worker	2960	54.6	94	50.3	1751	32.6	27	29.7
Self-employed	1768	32.6	55	29.4	1645	30.6	28	30.8
Agriculture or forestry	499	9.2	27	14.4	496	9.2	11	12.1
No occupation	199	3.7	11	5.9	1487	27.6	25	27.5
Marital status								
Married	4601	86.7	155	84.2	4483	80.2	67	71.3
Other than the above	708	13.3	29	15.8	1106	19.8	27	28.7
Educational background								
Junior college, college, or higher								
	832	15.3	16	8.2	382	6.7	4	4.0
Other than the above	4601	84.7	178	91.8	5290	93.3	95	96.0

**Table 2.** Baseline characteristics and numbers of deaths of men and women.

Characteristic	Men				Women			
	
	N	%	No. of deaths	%	N	%	No. of deaths	%
BMI, Body mass index								
BMI<9	343	6.2	32	16.2	450	7.7	17	17.3
19≦BMI<22	1641	29.5	58	29.4	1817	31.2	24	24.5
22≦BMI<25	2347	42.2	70	35.5	2215	38.0	27	27.6
25≦BMI<27	808	14.5	24	12.2	774	13.3	14	14.3
27≦BMI	418	7.5	13	6.6	574	9.8	16	16.3
Smoking								
Never	1407	25.9	38	20.1	4921	85.9	77	80.2
Former	930	17.1	40	21.2	117	2.0	3	3.1
Current								
<20 cigarettes/day	878	16.2	48	25.4	416	7.3	11	11.5
≧20 cigarettes/day	2209	40.7	63	33.3	273	4.8	5	5.2
Alcohol drinking								
Never	1200	22.0	62	33.2	3327	57.6	60	62.5
Light	2810	51.4	84	44.9	2302	39.9	34	35.4
Heavy	1454	26.6	41	21.9	145	2.5	2	2.1
Physical activity								
Never	2965	54.3	113	59.5	3445	59.7	62	65.3
Often, sometimes	2491	45.7	77	40.5	2326	40.3	33	34.7
Hours of sleep								
7-8 hours	3737	68.3	120	62.5	3639	62.9	66	67.3
>8 hours	657	12.0	44	22.9	315	5.4	15	15.3
<7 hours	1081	19.7	28	14.6	1832	31.7	17	17.3
Snacking								
Never	2811	51.1	86	44.1	1393	23.9	34	35.1
Often, sometimes	2688	48.9	109	55.9	4437	76.1	63	64.9
Skipping breakfast								
Never	4242	77.6	150	78.5	4490	77.9	77	81.1
Often, sometimes	1228	22.4	41	21.5	1275	22.1	18	18.9
Weight in 30’s								
Underweight	876	15.9	27	13.6	860	14.8	12	12.0
Average weight	3841	69.8	136	68.7	4006	69.1	66	66.0
Overweight	782	14.2	35	17.7	931	16.1	22	22.0
Perceived health status								
Excellent, good	1515	27.1	29	14.5	1260	21.4	16	15.8
Fair	3581	64.1	107	53.5	4180	71.0	61	60.4
Poor, very poor	488	8.7	64	32.0	450	7.6	24	23.8
Chronic diseases								
No	3567	65.2	78	40.6	3701	64.5	42	43.3
Yes	1901	34.8	114	59.4	2041	35.5	55	56.7
Health examination (in the past 3 years)								
No	821	15.2	56	29.3	981	17.4	38	40.4
Yes	4572	84.8	135	70.7	4659	82.6	56	59.6

The sociodemographic characteristics of the subjects are shown in Table 1. According to occupation, 54.6% of the men were salaried workers, and 9.2% were agriculture or forestry workers. The proportion of salaried workers among the women was 32.6%, while 27.6% had no occupation, and 9.2% were agriculture or forestry workers. More than 80% of both the men and the women responded that they were married.

Other characteristics are shown in Table 2. The proportion of subjects in the BMI category of 22 to less than 25 was the highest (about 40.0% of both men and women), the proportion with a BMI of 19 to less than 22 was second (about 30% of both men and women). The mean (± SD) of BMI was 23.0 (± 2.8) in the men and 23.0 (± 3.1) in the women.

The proportion of men who were current smokers and former smokers was 56.9% and 17.1%, respectively, and 12.1% and 2.0%, respectively, among the women. The proportion of heavy drinkers or light drinkers was 78.0% among the men and 42.4% among the women. The proportion of subjects who reported that their weight in their 30’s was overweight was 14.2% among the men and 16.1% among the women.

The proportion of subjects who reported their perceived health status as “poor” or “very poor” was 8.7% among the men and 7.6% among the women. The proportion of subjects who had chronic diseases was 34.8% among the men and 35.5% among the women.

### Survival status

A total of 304 persons (2.6%), 201 men (3.6%) and 103 women (1.7%), died, during the period 1993-1998 and a total of 110 persons (1.0%), 62 men (1.1%) and 48 women (0.8%), were lost to follow-up. The mean observation period was 5.1 years in Komochi Village and 4.3 years in Isesaki City. Eighty-one men (3.5%) and 37 women (1.7%) in Komochi Village, and 120 men (3.6%) and 66 women (1.8%) in Isesaki City died, and 9 men (0.4%) and 11 women (0.5%) in Komochi Village, and 53 men (1.6%) and 37 women (1.0%) in Isesaki City were lost to follow-up.

The proportion of men who died in both study areas was almost twice the proportion of women who died. The proportion of subjects lost to follow-up was small in both study areas. The proportion of men lost to follow-up was as almost same as the proportion of women.

### Causes of death

The causes of death in both men and women are shown in Table 3. Among men, the cause of death was malignant neoplasms in 82 (40.8%), heart disease in 35 (17.4%), cerebrovascular disease in 17 (8.5%). Among women, the cause of death was malignant neoplasms in 38 (36.9%), cerebrovascular disease in 18 (17.5%), and heart disease in 13 (12.6%). These three causes of death accounted for about 67% of all deaths in both men and women.

**Table 3.** Causes of death in men and women .

Cause of death	Men			Women		
	
	N	%	SMR	N	%	SMR
Malignant neoplasms	82	40.8	0.93	38	36.9	0.79
Heart diseases	35	17.4	1.36	13	12.6	1.28
Cerebrovascular diseases	17	8.5	0.83	18	17.5	1.61
Others	67	33.3		34	33.0	

All causes	201	100.0	1.09	103	100.0	1.13

SMR: Standard mortality ratio calculated by mortality according to sex and age category in Japan in 1997.

### All-cause mortality

Age-adjusted relative risks and multivariate relative risks are shown in Table 4.

*Age-adjusted relative risks:* Among the lifestyle factors, the BMI of men showed an L-shaped relationship (i.e., mortality increased at lowest level of the BMI spectrum) with age-adjusted relative risk for all-cause mortality, and the lowest relative risk was at a BMI of 22 to less than 25. Among women, the BMI showed a U-shaped relationship (i.e., mortality increased at both the highest and lowest level of the BMI spectrum) with age-adjusted relative risk for all-cause mortality, and the lowest relative risk was at a BMI of 22 to less than 25.

Among men, smoking and alcohol drinking were associated with all-cause mortality. Current smokers of fewer than 20 cigarettes per day had a significantly higher relative risk of death than nonsmokers, but current smokers of more than 20 cigarettes per day did not. Among men, light drinkers had a significantly lower relative risk of death than nondrinkers. Among women, smoking and alcohol drinking were not significantly associated with all-cause mortality, although tendencies similar to those seen in men were observed.

Both men and women who reported more than 8 hours of sleep a day had a significantly higher relative risk of death compared with the respondents reporting 7-8 hours of sleep. Men who skipped breakfast had a significantly higher relative risk of death compared with respondents who never skipped breakfast. Men who reported their weight as overweight in their 30’s had a significantly higher relative risk of death compared with respondents reporting their weight being underweight in their 30’s.

Both men and women who perceived their health status as poor or very poor had a significantly higher relative risk of death compared with those who perceived their health as excellent or good. Both men and women with chronic diseases had a significantly higher relative risk of death compared with those who did not have chronic diseases. Both men and women who had had a health exanimation in the past three years had a significantly lower relative risk of death than those who had never had a health exanimation.

Among the sociodemographic factors, being married and a junior college, college, or higher education were associated with significantly lower relative risk of death in men, but there were no significant associations in women. Occupation was not significantly associated with mortality in either men or women.

*Multivariate relative risks*: Multivariate relative risks adjusted for all variables (in Table 4) and the variable for study areas also showed tendencies similar to those of age-adjusted relative risks. BMI, weight in the subjects’ 30’s, and having had a health examination in the past three years were significantly associated with the mortality risk among both men and women. In addition, poor or very poor perceived health status was significantly associated with mortality risk in men, but not in women. Chronic diseases showed no significant association with the multivariate-adjusted relative risk.

BMI showed an L-shaped or U-shaped relationship with multivariate relative risks for all-cause mortality, with the lowest relative risks at a BMI of 22 to less than 25 in both men and women. The multivariate relative risk of dying among men with a BMI less than 19, 19 to less than 22, 22 to less than 25, 25 to less than 27, and 27 or more was 1.00 (referent), 0.57 (95%CI: 0.33-0.98), 0.48 (95%CI: 0.27-0.83), 0.54 (95%CI: 0.28-1.07), and 0.52 (95%CI: 0.24-1.14), respectively. Among women, the respective values were 1.00 (referent), 0.41 (95% CI: 0.20-0.85), 0.28 (95%CI: 0.13-0.60), 0.39 (95%CI: 0.16-0.92), and 0.45 (95%CI: 0.18-1.13), respectively.

The multivariate relative risk of being overweight in the subjects’ 30’s was 2.42 (95%CI: 1.28-4.58) among men and 2.75 (95%CI: 1.12-6.78) among women, compared with being underweight. The multivariate relative risk of having had a health exanimation in the past three years was 0.49 (95%CI: 0.33-0.74) among men and 0.46 (95%CI: 0.27-0.78) among women, and the risk of poor or very poor perceived health status among men was 4.55 (95% CI: 2.50-8.30).

## DISCUSSION

The authors conducted a population-based cohort study to assess the effects of lifestyle, social network, and physical/mental symptoms on mortality in middle-aged and elderly community residents. The questionnaire items covered lifestyle, psychosocial factors, including social network, and physical/mental symptoms. Some items included in the questionnaires were similar to those in the Alameda County Study, such as seven health practices and social network^2^^)^.

### Bias in the sample

The response rate of 91.6% in this study was rather high, and thus the non-response bias seems to have been small. Selection bias in this survey also did not seem large, because the mean BMI of the respondents and the smoking rate were almost as same as reported in the National Nutrition Survey, Japan, of 1993^13^^)^. In addition, as shown in Table 3, the calculated standard mortality ratio (SMR) of all-causes of death was 1.09 among the men and 1.13 among the women, indicating that the mortality in this survey was almost the same as in the total Japanese population.

### The observation period and losses to follow-up

Although the observation period in Isesaki City was 9 months shorter than in Komochi Village, the proportions of death in both areas were almost same. This finding may be reflected by differences in the average age in the two areas. The average age in Komochi Village was lower than in Isesaki City. The average age of the men and women in Komochi Village was 52.7 years and 53.1 years, as opposed to 54.9 years and 55.3 years in Isesaki City, respectively.

In this survey, there is no significant difference between respondents who were retained in the study and those who were lost to follow-up, in the characteristics such as sex, BMI, and chronic diseases, except the average age (respondents who were retained in the study: 54.3 years vs. those who were lost to follow-up: 48.1 years). Loss of a substantial number of subjects to follow-up raises serious doubts about the validity of a study^14^^)^. In this survey, fewer than 1-2% persons were lost to follow-up in either Komochi Village or Isesaki City, and thus the effect of losses to follow-up was considered to be small.

### The endpoint variables in the Cox proportional-hazards model

The authors examined not only all-cause mortality but cause-specific mortality (Table 3). Since the numbers of deaths in each category of cause-specific mortality were not very large, we used all-cause mortality alone as the endpoint variable in the hazards model for assessing risk factors.

### Effects of lifestyle factors on mortality

In the Alameda County Study seven lifestyle factors were shown to be risks for mortality: smoking, being obese or underweight, consuming excessive quantities of alcohol, being physically inactive, sleeping fewer than 7 or more than 8 hours per night, not eating breakfast regularly, and snacking^2^^)^. In this study, the variables having high risk of death (age-adjusted risk) were smoking, BMI, hours of sleep, and not eating breakfast, among the men, and BMI and hours of sleep, among the women. After multivariate adjustment, the only one of seven lifestyle factors in this study regarded as a risk factor for death was BMI in men and women.

### Obesity

The relation between body weight and mortality remains unclear, particularly with respect to optimal weight for longevity^15^^)^. Although the health hazards of severe obesity are well known in Western countries, the health consequences of being mildly to moderately overweight remain a matter of controversy. Also, some researchers have reported that leanness increases the risk of mortality, while other researchers have stated that the risk was no longer evident after appropriate control of confounding factors such as smoking in the analysis^15^^, ^^16^^)^. Many prospective studies have reported that the relationship between BMI and mortality is represented by a J- or U-shaped curve or is linear^15^^-^^21^^)^.

The curve for the relationship between BMI and mortality in this study was L-shaped in men and U-shaped in women, with the lowest mortality risk at a BMI of 22 to less than 25 for both men and women. These relationships remained after adjustment for age, smoking status, alcohol drinking, chronic disease, and the other risk factors shown in Table 4, suggesting that BMI is associated with mortality independent of these other risk factors.

**Table 4.** Relative risks of death from all causes in men and women.

Variable	Men				Women			
	
	Age-adjusted RR (95%CI)		Multivariate RR* (95%CI) (139 deaths/4500)		Age-adjusted RR (95%CI)		Multivariate RR* (95%CI) (71 deaths/4400)	
Age	1.11	(1.09-1.13)†	1.10	(1.07-1.13)	1.08	(1.06-1.11)†	1.06	(1.02-1.10)
BMI, Body mass index								
BMI<19	1.00		1.00		1.00		1.00	
19≦BMI<22	0.45	(0.29-0.69)	0.57	(0.33-0.98)	0.41	(0.22-0.76)	0.41	(0.20-0.85)
22≦BMI<25	0.39	(0.25-0.59)	0.48	(0.27-0.83)	0.33	(0.18-0.61)	0.28	(0.13-0.60)
25≦BMI<27	0.42	(0.25-0.71)	0.54	(0.28-1.07)	0.45	(0.22-0.92)	0.39	(0.16-0.92)
27≦BMI	0.46	(0.24-0.88)	0.52	(0.24-1.14)	0.72	(0.37-1.44)	0.45	(0.18-1.13)
Smoking								
Never	1.00		1.00		1.00		1.00	
Former	1.37	(0.88-2.14)	1.10	(0.66-1.84)	1.56	(0.49-4.95)	1.50	(0.36-6.22)
Current								
<20 cigarettes/day	1.72	(1.12-2.64)	1.44	(0.86-2.40)	1.65	(0.88-3.10)	1.46	(0.67-3.16)
≧20 cigarettes/day	1.28	(0.85-1.91)	1.05	(0.66-1.68)	1.49	(0.60-3.69)	1.13	(0.39-3.24)
Alcohol drinking								
Never	1.00		1.00		1.00		1.00	
Light	0.64	(0.46-0.89)	0.92	(0.61-1.37)	0.91	(0.60-1.39)	1.08	(0.66-1.78)
Heavy	0.71	(0.47-1.05)	1.01	(0.62-1.64)	1.10	(0.27-4.53)	1.19	(0.27-5.16)
Physical activity								
Never	1.00		1.00		1.00		1.00	
Often, sometimes	0.78	(0.59-1.05)	1.03	(0.73-1.46)	0.70	(0.46-1.07)	0.86	(0.52-1.42)
Hours of sleep								
7-8 hours	1.00		1.00		1.00		1.00	
>8 hours	1.52	(1.07-2.16)	1.50	(0.98-2.30)	1.99	(1.13-3.51)	1.45	(0.70-3.02)
<7 hours	0.93	(0.61-1.40)	0.93	(0.57-1.50)	0.59	(0.35-1.01)	0.40	(0.20-0.80)
Snacking								
Never	1.00		1.00		1.00		1.00	
Often, sometimes	1.26	(0.95-1.67)	1.41	(0.99-2.01)	0.65	(0.43-0.99)	0.70	(0.42-1.16))
Skipping breakfast								
Never	1.00		1.00		1.00		1.00	
Often, sometimes	1.55	(1.09-2.21)	1.18	(0.76-1.83)	1.03	(0.62-1.73)	0.75	(0.40-1.43)
Weight in 30’s								
Underweight	1.00		1.00		1.00		1.00	
Average weight	1.19	(0.78-1.79)	1.63	(0.96-2.76)	1.24	(0.67-2.30)	1.85	(0.85-4.03)
Overweight	1.66	(1.01-2.75)	2.42	(1.28-4.58)	1.90	(0.94-3.83)	2.75	(1.12-6.78)
Perceived health status								
Excellent, good	1.00		1.00		1.00		1.00	
Fair	1.55	(1.03-2.34)	1.22	(0.75-1.98)	1.02	(0.59-1.78)	0.82	(0.44-1.52)
Poor, very poor	6.58	(4.24-10.21)	4.55	(2.50-8.30)	3.78	(2.01-7.13)	1.82	(0.78-4.29)
Chronic diseases								
No	1.00		1.00		1.00		1.00	
Yes	2.06	(1.54-2.76)	1.05	(0.70-1.58)	1.91	(1.27-2.88)	1.15	(0.67-1.96)
Health examination (in the past 3 years)								
No	1.00		1.00		1.00		1.00	
Yes	0.46	(0.33-0.63)	0.49	(0.33-0.74)	0.30	(0.20-0.45)	0.46	(0.27-0.78)
Occupation								
Salaried worker	1.00		1.00		1.00		1.00	
Self-employed	0.87	(0.63-1.22)	0.63	(0.42-0.94)	1.01	(0.59-1.71)	0.75	(0.41-1.37)
Agriculture or forestry	1.01	(0.66-1.57)	0.50	(0.26-0.96)	0.84	(0.41-1.72)	0.74	(0.28-1.96)
No occupation	0.89	(0.47-1.68)	0.57	(0.28-1.18)	0.80	(0.46-1.39)	0.60	(0.32-1.13)
Marital status								
Married	0.62	(0.42-0.92)	0.74	(0.45-1.21)	0.77	(0.49-1.21)	0.74	(0.43-1.27)
Other than the above	1.00		1.00		1.00		1.00	
Educational background								
Junior college, college, or higher								
	0.59	(0.35-0.98)	0.75	(0.43-1.30)	0.81	(0.30-2.22)	0.82	(0.25-2.67)
Other than the above	1.00		1.00		1.00		1.00	

RR, Relative risk; CI, Confidence interval. RRs are based on the Cox proportional-hazards model.

* Adjusted for study areas (Komochi Village and Isesaki City) and listed all variables.

† The age-adjusted RR of age is from a model in which only age was entered.

In Western countries, higher BMIs are associated with higher mortality^15^^-^^20^^)^. However, as mentioned above, mortality did not significantly increase at higher BMI levels in this Japanese sample, and the same findings regarding the relationship between BMI and all-cause mortality have been reported in previous Japanese population-based studies^22^^, ^^23^^)^. For example, Ishii et al.^22^^)^ reported an L-shaped relationship in men with all-cause mortality being the lowest at BMI 24-26, and a U-shaped relationship in women with the lowest all-cause mortality at BMI 22-24. In the Hisayama Study, the authors reported a U-shaped relationship with the lowest mortality at a BMI of 23-27 in men and at a BMI of 23-25 in women^23^^)^. This may be attributable to the very small percentage of the Japanese population that is severely obese (BMI ≧ 30.0) compared with Western countries. The prevalence of obesity (BMI ≧ 30.0) in the National Health and Nutrition Examination Surveys (NHANES III: 1988-94) in the United States was 19.9% among men and 24.9% among women^24^^)^, whereas, in this study it was only 1.3% among men and 2.2% among women.

Furthermore, in this study the leanest group (BMI<19) of both men and women was considered to be at higher risk of death. This may be because of weight loss due to subclinical health conditions. Although the subjects were asked about their present health condition, including chronic diseases, that may not be an adequate means of assessing subclinical conditions of which the subjects were not aware. It is possible that some of the subjects who died in the leanest group may have had subclinical diseases related to weight loss at baseline. However, Nakayama et al.^23^^)^ reported that an elevated mortality risk was clearly observed in the leanest group after 10 years of follow-up. They suggested that leanness itself is a risk factor for mortality in the Japanese population, because the effects of subclinical health conditions cannot last for ten years. Since the observation period in the present study was only five years, we may have to wait some time before drawing any conclusions on this issue.

In this study, obesity in the subjects’ 30’s was also associated with a higher mortality risk. Previous studies have reported that past obesity was associated with increased risk of coronary heart disease, stroke, and mortality^25^^-^^27^^)^, and that weight history in middle age is important in determining the risk of disease^25^^-^^27^^)^. Our findings suggest that maintaining weight within normal range is important in early middle age.

### Smoking and alcohol drinking

In this study, male current smokers of fewer than 20 cigarettes per day had a significantly higher age-adjusted relative risk of mortality, but smokers of 20 cigarettes or more per day did not. In previous reports mortality risk increased with the amount smoked^28^^)^. This paradoxical finding may be explained by smokers with health problems already having reduced the number of cigarettes at baseline. This appears to be partly supported by the proportion of smokers of 20 cigarettes or more per day among men with chronic diseases being 34.2%, as opposed to 44.5% among men with no chronic diseases (data not shown).

In this study, the age-adjusted mortality risk of light drinkers among men was lower than among non-drinkers, however, after multivariate adjustment for both smoking and alcohol drinking, the RR was no longer significant. We will be able to estimate the mortality risk of smoking and alcohol drinking after a greater number of deaths have occurred. We may also have to wait to assess the mortality risk of smoking and alcohol drinking in women, because the proportions of smokers and heavy drinkers were too small.

### Eating breakfast, snacking, and hours of sleep

In this study, men who skipped breakfast had a higher age-adjusted relative risk than those who ate regularly, but no significant difference was found in the multivariate analysis. Of the seven lifestyle factors in the Alameda County Study, not eating breakfast regularly and snacking did not carry as important a mortality risk as the other five health practices^2^^)^. Our findings were similar to those in previous reports.

Extremely longer sleeping hours among men and women increased age-adjusted mortality risk in this study. This finding has been reported in the previous studies^2^^)^.

### Effects of perceived health and health behavior on mortality

In this study poor perceived health status was significantly related to higher mortality risk among men in both the age-adjusted analysis and multivariate analysis after adjusting for chronic diseases and other risk factors. Many previous reports suggested that people who assessed their health as poor had an increased relative risk of mortality compared to those who reported that they were in good health, even after age, chronic diseases, health practices, and demographic factors were controlled^6^^, ^^29^^-^^32^^)^. It remains unclear whether perceived health status depends on objective health status or not, because subjects who had subclinical disease may assess their perceived health as poor. Furthermore, some previous reports have suggested that perceived health is associated not only with objective health status, such as chronic diseases but with morale, social activity^32^^, ^^33^^)^, and psychological factors, such as depressive mood^34^^)^. These factors may affect mortality if perceived health status itself is a risk factor.

The association between perceived health status and mortality in this study was weaker in women than in men. Several studies have shown a greater contribution of perceived health status to mortality in men than in women^30^^, ^^32^^)^. Haga et al.^32^^)^ suggested that perceived health among women is more affected by objective health status, and thus the effect on mortality risk might be weaker after controlling for objective health status, such as chronic diseases.

Men and women who had undergone a health examination in the past three years had a lower mortality risk in both the age-adjusted and multivariate analysis. This finding has been reported in an earlier study^35^^)^. This is probably because people who have severe diseases tend not to receive health examinations since they are already attending a clinic^35^^)^, or participants in health examinations have a more positive behavior pattern in regard to health^36^^)^. However, the possibility remains that preventive health measures such as health examinations may contribute to reducing the mortality.

### Effects of sociodemographic factors on mortality

Men who were married had a lower age-adjusted morality risk. Previous reports have shown that being unmarried was associated with higher mortality of all causes^2^^, ^^37^^)^. Men who had a higher level of education had a lower age-adjusted mortality risk. Earlier studies have also reported that higher education level is associated with lower mortality^37^^, ^^38^^)^.

### Limitations of the study

Our research has several limitations. First, the baseline questionnaires were just for lifestyle and sociodemographic factors, not including physical and laboratory data in this study. Second, although we attempted to adjust the effects of confounding factors, such as chronic diseases, in this study, by using multivariate analysis, other confounding factors may still have been present. As mentioned earlier in this paper, one of the confounding factors was a subclinical condition, such as latent cancer, and the five-year follow-up period may have been insufficient to overcome bias due to subclinical conditions. It is possible that subclinical diseases at baseline may influence both lifestyle factors and mortality. Therefore, we repeated analyses excluding those who died within first one-year. The results were almost the same as presented above (data not shown). Third, the authors did not stratify the data by the study areas (Komochi Village and Isesaki City), because the numbers of deaths were not large for the stratification. Then, we include the variable for the study areas in the multivariate model. Fourth, self-reported questions such as alcohol drinking may have introduced some inaccuracy, though the authors tried to validate self-reported alcohol drinking by interview. “Heavy drinkers” and “light drinkers” corresponded mostly to “drink 2-3 *gou*/day” and “drink 1-1.9 *gou*/day”, respectively (data not shown). One *gou* (a Japanese traditional unit of *sake*) is equivalent to about 25 g of ethanol.

Despite these limitations, our study provides preliminary findings on risk factors for all-cause mortality in middle-aged and elderly community residents. Our findings suggested that increased risk of death was associated with a lower BMI, obesity in the subjects’ 30’s among both men and women, and poor perceived health status among men; on the other hand, decreased risk of death was associated with a moderate BMI (the lowest risk of death was shown at a BMI of 22-25) and undergoing health examinations among both men and women. Since we plan to conduct a follow-up questionnaire survey on this cohort in 2000-2001, these findings will allow us to decide which risk factor variables for mortality are important for the new questionnaires. Some people may change their lifestyle during the extended follow-up period, and we will be able to evaluate the association between mortality and lifestyle and other risk factors, taking account of such changes in future analyses.
